# Variation in classification of live birth with newborn period death versus fetal death at the local level may impact reported infant mortality rate

**DOI:** 10.1186/1471-2431-14-108

**Published:** 2014-04-22

**Authors:** Charles R Woods, Deborah Winders Davis, Scott D Duncan, John A Myers, Thomas Michael O’Shea

**Affiliations:** 1 Department of Pediatrics, University of Louisville School of Medicine, 571 S. Floyd Street, Suite 412, Louisville, KY, USA; 2 Department of Pediatrics, Wake Forest School of Medicine, Winston-Salem, NC, USA

**Keywords:** Fetal death, Infant mortality, Perinatal death, Birth classification

## Abstract

**Background:**

To better understand factors that may impact infant mortality rates (IMR), we evaluated the consistency across birth hospitals in the classification of a birth event as either a fetal death or an early neonatal (infant) death using natality data from North Carolina for the years 1995–2000.

**Methods:**

A database consisting of fetal deaths and infant deaths occurring within the first 24 hours after birth was constructed. Bivariate, followed by multivariable regression, analyses were used to control for relevant maternal and infant factors. Based upon hospital variances, adjustments were made to evaluate the impact of the classification on statewide infant mortality rate.

**Results:**

After controlling for multiple maternal and infant factors, birth hospital remained a factor related to the classification of early neonatal versus fetal death. Reporting of early neonatal deaths versus fetal deaths consistent with the lowest or highest hospital strata would have resulted in an adjusted IMR varying from 7.5 to 10.64 compared with the actual rate of 8.95.

**Conclusions:**

Valid comparisons of IMR among geographic regions within and between countries require consistent classification of perinatal deaths. This study demonstrates that local variation in categorization of death events as fetal death versus neonatal death within the first 24 hours after delivery may impact a state-level IMR in a meaningful magnitude. The potential impact of this issue on IMRs should be examined in other state and national populations.

## Background

The definition of the infant mortality rate (IMR) as the number of deaths in the first year after birth per 1000 live births gained popular acceptance by the late 1800’s [1]. As early as the 1920’s, public health officials proclaimed that a valid measure of the IMR was a necessary precursor to initiating strategies for reducing infant death rates [1]. Subsequently, the IMR has served in the following capacities: 1) as an indicator of the health of populations and to compare health and health care systems between nations and between subunits of nations; 2) to inform the development of public policy and programs aimed at improving the health of infants and childbearing women; 3) to identify health disparities and factors that contribute to poor pregnancy outcome; 4) as an outcome measure for program evaluation; and 5) to identify emerging trends [2-4].

Disparities in the birth rates and newborn care of infants, especially preterm infants, may lead to incongruent comparisons. Very early preterm infants have much higher neonatal mortality rates than do term and near-term live-born infants [5]. Differences in birth rates of very preterm infants can lead to substantial differences in unadjusted IMRs across demographic groups or regions [5-10]. Approaches to birth classification, resuscitation, and care of the extremely preterm infant may alter outcome and influence the IMR [11].

A consistent classification of perinatal deaths is necessary if IMR-based comparisons are to be meaningful. The World Health Organization definition of a live birth is “the complete expulsion or extraction from its mother of a product of conception, irrespective of the duration of the pregnancy, which, after such separation, breathes or shows any other evidence of life (e.g. beating of the heart, pulsation of the umbilical cord or definite movement of voluntary muscles - whether or not the umbilical cord has been cut….)” [12].

Even with this stringent definition, differences in reporting fetal and infant deaths continue. The landmark study, “Five Decades of Missing Females in China,” was among the first to highlight a bias in reporting of infant deaths [13]. Inaccurate reporting of infant births and deaths plagues statistical comparisons among very preterm infants [3,7,14-17]. Variations in assigning and reporting infant deaths may result in misleading comparisons at an international, national, regional or local level.

A recent outcomes study of births weighing less than 500 grams showed substantial variation in the proportion classified as neonatal death versus fetal death at the state level in the United States from 1999 through 2002 [3,18]. We hypothesize that systematic variation exists in the classification of neonatal death compared to fetal death and that the type and location of the hospital contributes to the variation. We evaluated this variation at the local level within a single state, North Carolina, from 1995 through 2000 to demonstrate the potential impact of such variation on state-level IMR. Prenatal and delivery room care of fetuses and newborns at the border of viability has been largely unchanged since the years in which the study data were collected, and no change has been made in definitions for fetal and infant death since that time.

## Methods

### Construction of the database and derived variables

Live birth, infant death, and fetal death files for North Carolina for the years of birth 1995–2000 were obtained from the North Carolina State Center for Health Statistics after approval of the university Institutional Review Board. These files are currently publicly available. This analysis used the subset of records that represented 1) fetal deaths and 2) infant deaths that occurred within the first 24 hours after live birth. The latter were identified by 1) information contained in two fields denoting *time lived* (one field listed the number of time units lived and the other unit of time (e.g. minutes, hours); and 2) comparing the calendar date of birth to the calendar date of death. The latter allowed 21 infant deaths to be classified as occurring within the first 24 hours after birth when data were missing in the *time lived* fields.

Four groups (see below) were determined after an initial view of the frequency distribution of the number of events per hospital for the 135 hospitals in the database, as we could not do a meaningful comparison of all hospitals due to sample size issues. A decision was made to retain 31 hospitals with larger sample sizes (60 or more events in the 6 years of data) and then group the other hospitals and situations into the three comparison groups for the 31 individual hospitals. It seemed rational to use birth events occurring outside as a distinct group. It also seemed reasonable to break the 104 hospitals with < 60 events during the study period into two groups as follows: 1) those in counties that contained one of the 31 ‘high event number’ hospitals and 2) those in counties that did not. We posited that there could be cross-coverage or other similarities in hospital culture within counties with more than one hospital. We had no way to confirm whether this was true. This decision was made prior to the performance of other analyses of association of these groups or hospitals or other covariates with the outcome.

North Carolina had 100 counties and 135 hospitals represented in the database during the study years. Individual hospitals were selected for this analysis if they had at least 60 birth events that were fetal deaths or infant deaths during the study period (Group 1). Three comparison groups were constructed from the remaining records: Group 2) fetal or infant delivery occurring outside of a hospital setting, regardless of county of occurrence; Group 3) fetal or infant delivery occurring in hospitals in counties where no hospital met the inclusion criterion of having at least 60 such events during the study period; and Group 4) fetal or infant delivery occurring in hospitals with less than 60 such events in counties with 1 or more hospitals having 60 or more such events.

To allow adjustment for potential differences in numbers and types of high-risk pregnancies managed among the hospitals, birth certificate data were used to construct categorical variables for birth year, birth weight, gestational age, gender of the fetus or infant, maternal race/ethnicity, delivery method, plural birth, prenatal care visits, maternal age, maternal education, alcohol use during pregnancy, tobacco use during pregnancy, prior fetal deaths or pregnancy terminations of any type, maternal history of the death of a prior live-born child, gravidity, parity, and marital status. Dichotomous variables were constructed for 1) occurrence of an adverse event during labor or delivery (e.g., fever, anesthetic complications, abruptio placenta, breech presentation, cord prolapse, fetal distress); 2) maternal medical history positive for a disease or predisposing condition (e.g. anemia, diabetes, hypertension, incompetent cervix, previous preterm delivery); and 3) presence of any congenital anomaly.

### Outcomes measure and statistical methods

The outcome measure used was whether a pregnancy outcome was classified as a fetal death or a live birth with infant death occurring within 24 hours after birth. The null hypothesis was that *hospital of birth* is not associated with this classification. Bivariate associations were evaluated using Pearson Chi square tests. Cramer’s V was used to assess correlation between two nominal variables (maternal county of residence and birth hospital). Two-level logistic regression modeling (one-stage clustering sampling frame) using a general estimating equations approach was used to determine variation among individual hospitals relative to control groups while adjusting for other predictor variables and the potential cluster effect of birth hospitals (i.e., correlation between outcomes for events within the same hospital). SPSS 22.0 (IBM SPSS Inc., Armonk, NY) was used for all analyses.

### Adjustment of reported deaths and live births for hospital variance impact on statewide IMR

Reported infant deaths and live birth files were used to determine initial numerators and denominators for IMRs. As the ratios of infant deaths within 24 hours after birth to fetal deaths were adjusted to selected reference standards, appropriate adjustments in numerators and denominators were made (addition or subtraction, depending on the number of fetal deaths reclassified as live births, and vice versa).

## Results

**Figure 1:**
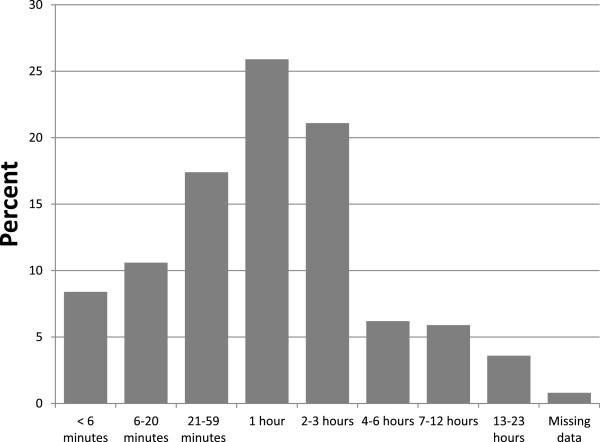
Time of death for 2733 infants dying within 24 hours after birth. Percentages of early neonatal (infant) deaths by time intervals after birth.

During the six years of 1995–2000 in North Carolina, there were a total of 649,252 live births, with 5813 infant deaths (8.95 per 1000 live births), and 5311 fetal deaths. Among the infant deaths, 2733 occurred within 24 hours after birth, with 89.7% occurring during the first six hours after birth (Figure 1). The population of pregnancies with outcomes classified as either fetal deaths or *early neonatal* deaths within the first 24 hours after birth consisted of 8044 such events.

### Factors associated with classification of pregnancy outcome

Twelve factors were associated with classification as a fetal death or early neonatal deaths within the first 24 hours after birth (Table 1). These included maternal race/ethnicity, birth weight, gestational age, method of delivery, maternal history of medical or predisposing conditions, presence of any congenital anomaly, number of prenatal care visits, maternal age, maternal education, plural birth, birth hospital, and maternal county of residence (data not shown in table). Relative to a reference group of 70 counties each with < 1% of the statewide births during the study period, odds ratios among the 30 counties with larger contributions to statewide births varied 3.3-fold (0.51 to 1.69) in the probability of pregnancy outcomes being classified as early neonatal versus fetal deaths.

**Table 1:** Classification of fetal death relative to infant death among reported live births living less than 24 hours and fetal deaths, North Carolina, 1995-2000

**Characteristic/factor**	**Bivariate associations**						**Multivariable associations‡**			
		**Fetal death**		**Infant death**						
	Total*	**N**	**%**	**N**	**%**	**Odds ratio†**	**Total**	**Odds ratio†**	**95% C.I. §**	**P ** **value**
**Maternal race/ethnicity**										
Other/Unknown	258	161	62.4	97	37.6	1.53	240	1.28	0.86 – 1.90	.23
White	3688	2511	68.1	1177	31.9	1.19	3511	1.13	0.87 – 1.47	.35
Black	3616	2293	63.4	1323	36.6	1.47	3488	1.30	1.00 – 1.07	.050
Latino	482	346	71.8	136	28.2	1	406	1		
**Birth weight (grams)**										
< 500	3130	1773	56.6	1357	43.4	4.54	2986	6.39	4.73 – 8.64	< .001
500-750	1454	755	51.9	699	48.1	5.49	1397	7.42	5.43 – 10.1	< .001
751-1000	491	356	72.5	135	27.5	2.25	464	2.47	1.72 – 3.54	< .001
1001-1500	546	430	78.8	116	21.2	1.60	519	1.39	0.97 – 2.01	.077
1501-1800	309	246	79.6	63	20.4	1.52	292	1.25	0.82 – 1.91	.30
1801-2000	202	148	73.3	54	26.7	2.16	192	1.93	1.23 – 3.03	.004
2001-2500	457	372	81.4	85	18.6	1.35	438	1.32	0.90 – 1.94	.16
2501-4000	942	792	84.1	150	15.9	1.12	904	1.18	0.84 – 1.66	.35
> 4000	513	439	85.6	74	14.4	1	413			
**Gestational age**							††			
< 24 weeks	3907	2083	53.3	1824	46.7	2.92				
24 weeks	437	230	52.6	207	47.4	3.00				
25 weeks	294	197	67.0	97	33.0	1.64				
26 weeks	230	181	78.7	49	21.3	0.90				
27 weeks	185	151	81.6	34	18.4	0.75				
28 weeks	205	165	80.5	40	19.5	0.81				
29-30 weeks	339	268	79.1	71	20.9	0.88				
31-32 weeks	420	343	81.7	77	18.3	0.75				
33-34 weeks	398	329	82.7	69	17.3	0.70				
35-36 weeks	441	364	82.5	77	17.5	0.71				
37-41 weeks	1051	881	83.8	170	16.2	0.64				
> = 42 weeks	26	20	76.9	6	23.1	1				
**Delivery method**										
Vaginal	7060	4777	67.7	2283	32.3	1	6691	1		
C-section	946	497	52.5	449	47.5	1.89	914	3.76	3.16 – 4.48	< .001
**Maternal medical history positive for diseases or predisposing conditions**										
No	4341	2949	67.9	1392	32.1	1	4091	1.09	0.97 – 1.20	.92
Yes	3703	2362	63.8	1341	36.2	1.20	3514	1		
**Presence of any congenital anomaly**										
No	7259	4902	67.5	2357	32.5	1	6880	1		
Yes	785	409	52.1	376	47.9	1.91	725	3.03	2.51 - .365	<.001
**Prenatal care visits**							††			
0-2 visits	1228	756	61.6	472	38.4	1				
3-7 visits	3251	1968	60.5	1283	39.5	1.04				
8-12 visits	2004	1450	72.4	554	27.6	0.61				
> = 13 visits	1005	725	72.1	280	27.9	0.62				
**Maternal age (years)**										
< 18	569	390	68.5	179	31.5	1.56	546	1.94	1.25 – 3.01	.003
18-19	807	518	64.2	289	35.8	1.90	785	2.25	1.49 – 3.40	< .001
20-24	2225	1435	64.5	790	35.5	1.88	2126	2.07	1.41 – 3.06	< .001
25-29	1975	1317	66.7	658	33.3	1.70	1870	1.76	1.19 – 2.59	.005
30-34	1441	907	62.9	534	37.1	2.01	1357	2.05	1.38 – 3.03	< .001
35-39	787	551	70.0	236	30.0	1.46	741	1.54	1.02 – 2.32	.041
> = 40	194	150	77.3	44	22.7	1	180	1		
**Maternal education**										
Beyond college	320	208	65.0	112	35.0	1.14	318	1.21	0.89 – 1.63	.23
College graduate	884	541	61.2	343	38.8	1.34	877	1.36	1.10 – 1.69	.005
1-3 years of college	1544	934	60.5	610	39.5	1.38	1530	1.35	1.13 – 1.62	.001
High school graduate	2941	1970	67.0	971	33.0	1.04	2920	1.02	0.87 – 1.19	.84
Less than high school	1976	1341	67.9	635	32.1	1	1960	1		
**Plural birth**										
No	7056	4825	68.4	2231	31.6	1	6653	1		
Yes	988	486	49.2	502	50.8	2.23	952	1.59	1.37 – 1.86	< .001
**Geographic variation, birth hospital**										
Births in smaller counties	1522	1087	71.4	435	28.6	1	1450	1		
Out of hospital births	140	95	67.9	45	32.1	1.18	118	2.29	0.99 – 5.27	.052
Low birth hospitals in larger counties	228	174	76.3	54	23.7	0.78	210	0.62	0.30 – 1.27	.19
01	63	54	85.7	9	14.3	0.42	63	1.40	0.36 - 5.35	.63
02	107	91	85.0	16	15.0	0.44	100	1.18	0.46 - 3.04	.73
03	76	61	80.3	15	19.7	0.61	76	0.56	0.14 - 2.29	.42
04	106	85	80.2	21	19.8	0.62	100	0.91	0.35 - 2.32	.84
05	214	167	78.0	47	22.0	0.70	209	**0.39**	0.18 - 0.84	.016
06	99	77	77.8	22	22.2	0.71	98	0.32	0.09 - 1.13	.08
07	106	82	77.4	24	22.6	0.73	106	0.96	0.40 - 2.33	.93
08	545	410	75.2	135	24.8	0.82	537	**0.54**	0.34 - 0.84	.007
09	86	64	74.4	22	25.6	0.86	81	0.17	0.02 - 1.13	.067
10	105	78	74.3	27	25.7	0.86	101	0.59	0.20 - 1.72	.33
11	76	56	73.7	20	26.3	0.89	76	0.23	0.05 - 1.20	.08
12	127	93	73.2	34	26.8	0.91	121	0.95	0.39 - 2.33	.90
13	205	150	73.2	55	26.8	0.92	189	1.43	0.77 - 2.67	.26
14	147	103	70.1	44	29.9	1.07	144	0.61	0.29 - 1.29	.20
15	293	202	68.9	91	31.1	1.13	257	0.91	0.51 - 1.62	.75
16	97	66	68.0	31	32.0	1.17	96	1.05	0.44 - 2.50	.91
17	65	44	67.7	21	32.3	1.19	61	0.75	0.18 - 3.10	.69
18	237	159	67.1	78	32.9	1.23	213	1.60	0.81 - 3.19	.18
19	87	58	66.7	29	33.3	1.25	80	1.46	0.54 - 3.97	.46
20	69	45	65.2	24	34.8	1.33	69	0.95	0.31 - 2.93	.93
21	110	70	63.6	40	36.4	1.43	110	1.14	0.49 - 2.64	.76
22	106	66	62.3	40	37.7	1.51	102	0.47	0.15 - 1.44	.18
23	117	72	61.5	45	38.5	1.56	115	0.56	0.23 - 1.39	.22
24	332	200	60.2	132	39.8	1.65	307	1.40	0.82 - 2.41	.22
25	356	214	60.1	142	39.9	1.66	352	**1.69**	1.06 - 2.70	.027
26	288	170	59.0	118	41.0	1.73	276	1.04	0.58 - 1.86	.90
27	73	43	58.9	30	41.1	1.74	73	2.49	0.96 - 6.43	.060
28	364	197	54.1	167	45.9	2.12	363	1.55	0.97 - 2.46	.066
29	515	274	53.2	241	46.8	2.20	419	**2.30**	1.50 - 3.54	< .001
30	555	289	52.1	266	47.9	2.30	534	**2.12**	1.45 - 3.11	< .001
31	428	215	50.2	213	49.8	2.48	399	**2.33**	1.50 - 3.60	< .001

*The total population of events was 8044. Four variables had missing data. Total records with data for these were: gestational age = 7933, prenatal care visits = 7488, maternal age = 7998, and maternal education = 7665.

† Odds of classification as infant (early neonatal) death compared to fetal death (reference group OR = 1). For bivariate associations, each listed variable had *p* < .001.

‡ There were 7605 records with data for the 9 variables included in the multivariable model. A logistic regression model using a one-stage cluster design (birth hospital) was used for this analysis.

§ C.I. = confidence interval.

†† Variable was not included in the modeling process (see text).

The following factors had neither meaningful nor statistical association with the classification outcome (all but one with *p* > .10): birth year, alcohol use during pregnancy, tobacco use during pregnancy, occurrence of an adverse event during labor or delivery, prior fetal deaths or pregnancy terminations of any type, maternal history of the death of a prior live-born child, gravidity, parity, marital status (*p* = .064), and gender of the fetus-infant.

**Figure 2:**
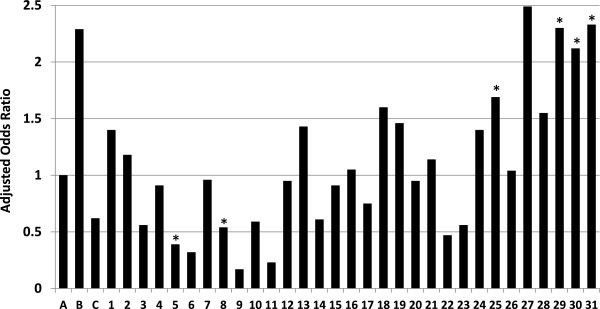
**Adjusted odds ratios of perinatal birth event classifications among the 31 hospitals and three control groups.** Adjusted odds ratios of the number of perinatal events classified as an early neonatal death (live birth followed by infant death occurring within 24 hours of birth) versus classified as a fetal death by three control groups and 31 individual hospitals with at least 60 such combined events during the study period. A = reference group of birth events in counties with small numbers of births. B = birth events that did not occur in a hospital. C = birth events in other hospitals in counties where one of the 31 individual hospitals was located. 1 – 31 = individual hospitals with ≥60 birth events during the study period. * = significantly different from the reference group (A).

Nine factors listed in Table 1 were evaluated in a one-stage cluster sampling frame logistic regression analysis modeling with birth hospital as the cluster variable. Seven of the nine, including birth hospital, had one or more subcategories that differed from the reference group (95% Confidence Interval excluded 1.0) with all variables entered. There was considerable variation among the 31 institutions compared to the reference group that pooled birth events in counties that did not have hospitals with large numbers of deliveries. Adjusted odds ratios among the six institutions that differed from the reference group varied 6-fold (0.39 to 2.33). Among all 31 hospitals evaluated individually, the variation was nearly 15-fold (.17 to 2.49). This variation is depicted in Figure 2. The three hospitals with statistically significant adjusted odds ratios >2.0 were each affiliated with a different academic medical center.

The strongest associations were seen with the lowest two birth weight groups, < 500 and 500–750 grams, which were 6.4 and 7.4 times as likely to be classified as early neonatal versus fetal deaths as those with birth weights > 4000 grams (Table 1). Significant, but smaller odd ratios were seen for infants weighing 751–1000 grams (2.47-fold; *p* < .001) and 1801–2000 grams (1.93-fold; *p* = .004) who were also more likely to be classified as early neonatal versus fetal deaths as birth weights > 4000 grams (Table 1). Infants who died within the first 24 hours who delivered by C-section were almost 4-fold as likely to be classified as infant deaths relative to those delivered vaginally. Those with congenital anomalies were 3-fold as likely to be categorized as early neonatal death than infants without anomalies. Plural birth events were 1.6-fold more likely to be classified as neonatal rather than fetal deaths.

Infants born to all maternal age groups < 40 years old were 1.5 to 2.3-fold more likely to be classified as neonatal deaths compared with those born to mothers who were ≥ 40 years old. (Table 1) Maternal education that included some college or college graduation, but not beyond a college degree, was associated with greater likelihood of neonatal versus fetal death classification relative to those who did not graduate from high school. Black race bordered on significance (odds ratio = 1.30, 1.00 – 1.070). Maternal medical history positive for diseases or predisposing conditions was not associated with birth outcome classification.

Gestational age, prenatal care visits, and maternal county of residence were not used in the final model. Gestational age and birth weight were highly correlated, with a Spearman correlation coefficient of 0.78 (*p* < .001). Birth weight was known for all 8044 cases, while gestational age was missing for 111 (1.4%). *Prenatal care visits* were missing from 556 cases (6.9%). The number of prenatal visits was modestly correlated with birth weight (Spearman correlation coefficient of 0.37, *p* < .001). Given scattered missing data in other variables, inclusion of *prenatal care visits* in the final model would have resulted in loss of > 10% of evaluable records.

Maternal county of residence also was excluded from multivariable analysis as this was highly associated with birth hospital (Cramer’s V coefficient = 0.71 for the birth events at the 31 individual hospitals, *p* < .001). A single county accounted for ≥80% of maternal residence for 14 (45%) of the 31 individually-evaluated hospitals. Two counties accounted for ≥90% of maternal residence for another 4 (13%) and for ≥75% for another 6 (19%).

### Impact of adjusted ratios on reported infant mortality rates

Inspection of the percentages of outcomes classified as fetal deaths and adjusted odds ratios relative to the reference group of hospitals in smaller population counties suggested four strata among the 31 hospitals (Tables 1 and 2). Eight hospitals comprised a group that classified at least 75% of events as fetal deaths. Another group of four, three of which were part of academic medical centers, classified < 55% as fetal deaths.

**Table 2:** Impact on reported statewide infant mortality rate for 1995–2000 if all hospitals classified events similarly according to each of four groups on percentage of events classified as fetal deaths

**Hospital group (N)**^**A**^	**Group definition**	**Events (%) within group classified as fetal death**	**Total events (%) in group**	**Infant nortality if all classified similarly to group**^**B**^
1 (8)*	≥75% of outcomes classified as fetal deaths	77.8%	1544 (19.2)	7.50
2 (15)^†^	61 -- 74.9% of outcomes classified as fetal deaths	69.9%	3589 (44.6)	8.48
3 (4)^‡^	55 – 60.9% of outcomes classified as fetal deaths	59.8%	1049 (13.0)	9.73
4 (4)^║^	<55% of outcomes classified as fetal deaths	52.4%	1862 (23.1)	10.64
Total	--	66.0%	8044	8.95^C^

^A^Number of the 31 hospitals selected for individual analysis based on ≥ 60 fetal death/early neonatal death events during the study period.

*This group also included events from hospitals with low birth numbers in counties with one of the 31 hospitals. This group contained the two hospitals with odds ratios that were statistically lower than the reference group.

^†^This group also included the reference group of hospitals in counties with < 1% of statewide births during the study period as well as the 140 deliveries that occurred outside of hospitals. All 15 hospitals in this group had 95% C.I.s of adjusted odds ratios that contained 1.0.

^‡^Three of these four hospitals had adjusted odds ratios ≥1.40, one of which was statistically higher than the reference group.

^║^Three of these four hospitals had adjusted OR >2.0 that were statistically higher than the reference group. These three were affiliated with different academic medical centers.

^B^Infant deaths per 1000 live births. Live birth denominator was adjusted for reclassification of fetal deaths as live births or live births as fetal deaths, as indicated. Total number of infant deaths reported in North Carolina from 1995–2000 was 5815. The adjusted number of infant deaths for the calculations of groups 1 through 4 was 4868, 5503, 6316, and 6911, respectively.

^C^Actual reported infant mortality rate for North Carolina, based on total reported infant deaths and live births for the six-year period.

To evaluate potential impact on state level IMR of the observed variation among hospitals in classification of these pregnancy outcomes as fetal deaths or infant deaths, the aggregate reported live births and infant deaths from 1995–2000 were used as starting points. If all hospitals statewide had classified these pregnancy outcomes similar to those in Group 1 with the highest fetal death percentage, the IMR for North Carolina during 1995–2000 would have been 7.5, which is 16% lower than the rate based on reported live births and infant deaths during this time. If all hospitals had classified outcomes similar to those in Group 4 with the lowest fetal death percentage, the IMR would have been 10.64, which is 19% higher than the rate based on reported live births and infant deaths during this time. There would have been a similar increase and decrease, respectively, in the reported fetal death rate during this time period.

## Discussion

In this study, the birth hospital was an important predictor of whether the death was classified as a fetal or infant death. Among the 31 hospitals selected for study, there was a nearly 15-fold variation in the probability of events being classified as early neonatal versus fetal death after controlling for numerous other factors that may be associated with this outcome. Had all hospitals in the state classified these birth events at similar low or high fetal death proportions based on the rates of the lowest and highest of four hospital-rate-strata, the aggregate IMR of North Carolina from 1995–2000 could have been adjusted from 16% lower to 19% higher than the reported 8.95/1000 (range approximately 7.5/1000 to 10.7/1000).

The IMR is a key measure of population health and is widely used as a comparative measure, determinate of healthcare policy, and/or an outcomes measure. Preterm birth and its complications are well-recognized causes of infant death. Differences in preterm birth rates and interventions have been identified as explanatory factors for apparent difference in IMR between populations. Further, differences in classification and reporting of infant or fetal deaths have also been suggested as a factor for differences in IMR among various entities or regions [17-21]. However, within-state differences have not been previously reported.

Of note, the three hospitals with statistically significant odds ratios of classifying these events as early neonatal deaths that were more than 2-fold higher than the reference group were affiliated with three different academic medical centers. This could reflect greater rigor in adhering to live-birth definitions in these centers, greater availability of resources to resuscitate and care for extremely low birth weight neonates, and/or other unrecognized factors at these institutions relative to other sites of newborn care.

Interventions at the limits of gestation may also vary based upon physician attitudes and parental preferences. Factors that have been implicated in interventions at the limits of viability include maternal age, parity, race, insurance status, education, prenatal care, gestational age, and birth weight [11]. These decisions are often made under inherently stressful circumstances for the affected family and the health care providers who must make the classification. The approach taken by a physician with end-of-life decisions may influence the reporting of fetal versus infant death.

For many obstetricians and neonatologists, uncertainty exists in decisions to intervene and/or resuscitate between 500–600 grams or 23–24 weeks gestation [22,23]. A preterm infant on the edge of viability may be less likely to be offered intubation and ventilation in the delivery room, compared to those infants of higher gestational ages [24]. Physician age and experience have been correlated with willingness to withhold or withdraw care; surprisingly, there is no association with working in a larger NICU or a teaching hospital [22,24,25]. Improved reporting of fetal death rates in recent years also has been associated with an increase in fetal deaths, especially at 20–22 weeks gestation, relative to total births [26].

Much of the relatively high IMR in the United States can be attributed to a high percentage of preterm births [9,15]. A recent analysis of fetal death rates and < 24-hour-post-delivery infant mortality rates for deliveries of infants weighing less than 500 grams found differing classification rates among individual states [18]. The authors of this study speculated that the state-level differences observed could result from variation in reporting practices of a few individual hospitals. Our analysis of data from North Carolina, while not restricted to this low birth weight stratum, supports this contention.

Variations in classification of fetal deaths and infant deaths on the first postnatal day could potentially misinform efforts to prevent adverse outcomes of pregnancy. Until recently, the focus in the U.S. has been more toward reducing infant mortality with less attention being given to the problem of fetal mortality. It is now clear that fetal mortality, even when limited to fetal death beyond 20 weeks gestation, is a significant problem and that it has been underreported [16,27]. Interventions to prevent fetal death likely differ from interventions to prevent infant death.

Our study was limited by the inability to ascertain directly whether any of the reported fetal deaths actually showed signs of life that would have met the WHO definition of live birth. However, the variation among birth hospitals persisted in two-level logistic regression modeling to control for potential unmeasured confounding at the hospital level as well as multiple other factors that may contribute to true fetal death versus true live birth with rapid demise. Our analysis also was restricted to rapid demise after birth, with 90% of infant deaths occurring within 6 hours after birth. These “very early neonatal deaths” and many fetal deaths reasonably can be construed as a clinical continuum “ready-made” for subjectivity in classification despite the extant international definition of live birth.

Additional limitations of our retrospective cohort study include lack of any data elements beyond those collected as part of the vital statistics programs for live births, fetal deaths, and infant deaths during the study period. Some of the captured data elements, such as self-reported alcohol use during pregnancy, are not always sensitive or accurate measures. We also are unable to account for any under-reporting of fetal deaths beyond 20 weeks gestation during the study period, though we believe this would have been, at most, a rare occurrence [17].

Lastly, the age of our data is the primary limitation, but we believe the point we are able to illustrate remains important. To the extent that delivery room care of fetuses and newborns at the border of viability changed after 2000, our data conceivably might not be relevant to current practice. However, because we are aware of no efforts at a state or national level to standardize classification of deaths at the border of viability in the United State, it is likely that our study demonstrates the potential impact of a variation in practice that still exists. Additionally, there have been no changes in national regulations for registration of stillbirths or live births in the U.S. in the past 20 years. The rates of live births and still births have declined slightly in recent years, corresponding with the economic downturn in the U.S., but we do not believe these changes would influence practice variation in classification of live birth versus fetal death status in the delivery rooms of most local hospitals. Even if the local hospital-level variation we detected in this study has declined during the subsequent decade, this type of variance, which has not been previously described, still could have relevance and should be considered in future comparative analyses of infant mortality and other birth-related vital statistics between states and nations.

Repeating this analysis in more current databases from other regions of the U.S. and other countries would add further insight regarding the importance of this issue on reported IMRs. Future research would be strengthened by the inclusion of a mixed-methods approach that adds qualitative data from health care providers and staff involved in delivery and newborn care to better understand origins of variation in classification by hospital or hospital type. This could lead to system-level interventions that improve adherence to the current definition of live birth and reduce variation in classification.

## Conclusions

The purpose in this analysis was to demonstrate that local hospital-level variation in classification of live birth with death in the newborn period versus fetal death may have an impact on reported IMR at the state level that is important both clinically and for policy development. Impacts at the state level could, in turn, impact national IMR. Vigilance and diligence at local and state levels are needed to ensure consistent classification of early neonatal deaths so that valid comparisons can be made between counties and states.

Integrity of international or intra-national state/provincial comparisons of IMR as a measure of population health might be improved if fetal and neonatal death rates were compared by birth weight and/or gestational age strata rather than single aggregate summary statistics. Our findings further support the utility of Perinatal Mortality as a metric, whether defined as stillbirths after 22 weeks gestation plus infant deaths within seven completed days after birth [28,29] or other variants such as fetal deaths at or beyond 20 weeks gestation plus infant deaths under age 28 days [30]. A combined fetal death plus newborn-period death metric also may have utility in comparing the health of populations or effectiveness of health care systems and should be further evaluated.

## Competing interests

We have no financial or non-financial competing interests to disclose.

## Authors’ contributions

CRW initiated the study, developed the analysis database, conducted most analyses, produced the initial draft of the manuscript, and supported the development of the final manuscript. He gives final approval for publication of the current version of the manuscript. DWD participated in interpretation of the data and development and ongoing revision of the manuscript. She gives final approval for publication of the current version of the manuscript. SDD participated in interpretation of the data and development and ongoing revision of the manuscript. He gives final approval for publication of the current version of the manuscript. JAM conducted analyses and supported development of the final manuscript. He gives final approval for publication of the current version of the manuscript. TMO participated in the study design, interpretation of data analyses, and revising the manuscript for important intellectual content. He gives final approval for publication of the current version of the manuscript.

## Pre-publication history

The pre-publication history for this paper can be accessed here:

http://www.biomedcentral.com/1471-2431/14/108/prepub
